# Supplementary material for: Time‑resolved multi-omic analysis of paclitaxel exposure in human iPSC‑derived sensory neurons unveils mechanisms of chemotherapy‑induced peripheral neuropathy
Source: Cell Death Dis. 2026 Feb 10;17(1):211. doi: 10.1038/s41419-026-08445-2 (PMC12921266; doi:10.1038/s41419-026-08445-2)
Supplement: Supplementary file 15 — Dataset 1. [file 41419_2026_8445_MOESM15_ESM.zip › CDD_Open_Data/04_RNA_Seq/Timeline_panel_plots_etc_2023-01-23_1609.zip!/Panel_Plots_etc/Evidence_Plots/post5d/evidence_plot_standard contrasts_msigdb_hallmark_5d_PTX_vs_DMSO_ID6_M5892_HALLMARK_CHOLESTEROL_HOMEOSTASIS.pdf]

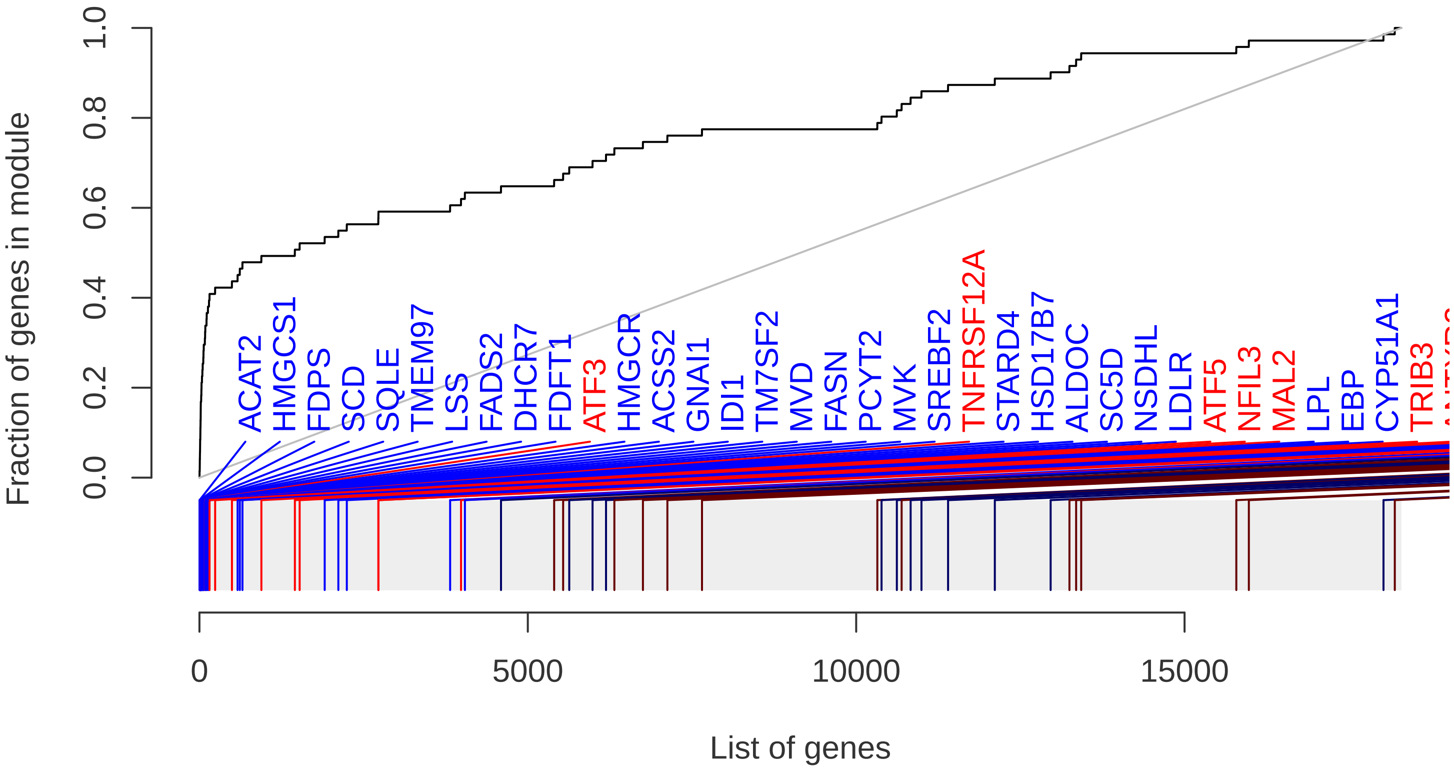

Fraction of genes in module
0.0
0.2
0.4
0.6
0.8
1.0
ACAT2
HMGCS1
FDPS
SCD
SQLE
TMEM97
LSS
FADS2
DHCR7
FDFT1
ATF3
HMGCR
ACSS2
GNAI1
IDI1
TM7SF2
MVD
FASN
PCYT2
MVK
SREBF2
TNFRSF12A
STARD4
HSD17B7
ALDOC
SC5D
NSDHL
LDLR
ATF5
NFIL3
MAL2
LPL
EBP
CYP51A1
TRIB3
0
5000
10000
15000
List of genes
